# Standardization of a Supramaximal Stimulation Protocol for Electrophysiological Assessment of the Intact Rat Median Nerve

**DOI:** 10.1155/bmri/1960458

**Published:** 2026-04-17

**Authors:** Tiago Bastos Taboada, Fernanda Marques Pestana, Julia Teixeira Oliveira, Geraldo Marco Rosa Junior, Henrique Rocha Mendonça, Beatriz Theodoro da Silva, Natália Tavares de Campos, Fernanda Martins de Almeida, Ana Maria Blanco Martinez

**Affiliations:** ^1^ Laboratory of Neurodegeneration and Repair, Department of Pathological Anatomy, Clementino Fraga Filho University Hospital, Federal University of Rio de Janeiro (UFRJ), Rio de Janeiro, Brazil, ufrj.br; ^2^ Graduate Program in Rehabilitation Sciences, School of Physiotherapy, Federal University of Rio de Janeiro (UFRJ), Rio de Janeiro, Brazil, ufrj.br; ^3^ Graduate Program in Pathological Anatomy, School of Medicine, Federal University of Rio de Janeiro (UFRJ), Rio de Janeiro, Brazil, ufrj.br; ^4^ Department of Anatomy, Dentistry School, Faculty of the Midwest Paulista (FACOP), Piratininga, São Paulo, Brazil; ^5^ Institute of Biomedical Sciences, Federal University of Rio de Janeiro (UFRJ), Rio de Janeiro, Brazil, ufrj.br

## Abstract

**Introduction:**

Electroneuromyography (ENMG) is the gold‐standard method for assessing peripheral nerve function through compound muscle action potential (CMAP) amplitude and latency. However, methodological heterogeneity across studies limits reproducibility and comparability, particularly regarding stimulation intensity and electrode configuration.

**Objective:**

This study is aimed at standardizing an ENMG protocol for the intact median nerve in rats by defining the minimal supramaximal stimulation intensity required to evoke maximal CMAP amplitude with stable latency.

**Methods:**

A total of 10 adult male Wistar rats (250–300 g) were anesthetized with ketamine and xylazine. The right median nerve was surgically exposed at the brachial plexus level. A bipolar hook electrode delivered square‐wave pulses (0.1 ms, 1 Hz) at descending intensities (20–0.1 V), with a 60‐s interval between intensity blocks. CMAPs were recorded from the flexor digitorum superficialis using monopolar needle electrodes. Signals were amplified (10 Hz–10 kHz band‐pass) and digitized at 20 kHz. Amplitude and latency were analyzed across intensities to determine the supramaximal plateau.

**Results:**

CMAP amplitude reached a plateau between 20 and 10 V, indicating full motor unit recruitment. Below 10 V, amplitude decreased progressively, and no response was observed at 0.1 V. Latency remained stable within the supramaximal range and increased only near threshold intensities.

**Conclusion:**

This study establishes a standardized supramaximal stimulation parameter (10 V) for electrophysiological assessment of the intact rat median nerve. Defining this reference intensity enhances methodological consistency and supports reproducibility in experimental models of peripheral nerve research.

## 1. Introduction

Peripheral nerves play a fundamental role in maintaining sensory and motor function, and their impairment can result in severe deficits affecting quality of life [1, 2]. The median nerve is particularly relevant for experimental research due to its mixed motor–sensory composition, anatomical accessibility, and translational importance in upper‐limb function across species [3–5]. Because of these characteristics, it serves as one of the most reliable models for investigating electrophysiological properties and testing new therapeutic interventions in peripheral nerve research.

Electroneuromyography (ENMG) is widely recognized as the gold‐standard method for evaluating peripheral nerve function. By recording compound muscle action potentials (CMAPs), ENMG provides quantitative information about axonal excitability, conduction velocity, and neuromuscular transmission integrity [5, 6]. CMAP amplitude reflects the number of activated motor units. Latency is defined as the time interval between the stimulus artifact and the onset of the CMAP [5]. It reflects the conduction time along motor fibers and, when interpreted together with conduction distance, serves as an indirect indicator of myelin integrity. [2, 7]. These parameters are critical for interpreting both physiological and pathological changes in peripheral nerve conduction.

Despite its extensive use in experimental and clinical settings, methodological heterogeneity remains a major obstacle to reproducibility in ENMG studies. Differences in electrode type and placement, stimulus intensity, filtering parameters, and recording sites can significantly alter CMAP amplitude and latency, thereby hindering cross‐study comparison [5, 6]. Standardization of these parameters in intact nerves is essential for providing reliable baseline data that can serve as physiological references in subsequent models of injury and repair [3, 4].

Previous studies in both rodents and primates have demonstrated that electrophysiological recordings of the median nerve are highly sensitive to axonal and myelin alterations following trauma or experimental manipulation [1, 2, 4]. However, few investigations have systematically characterized the intensity–response relationship in healthy animals. Determining the minimal supramaximal stimulus required to achieve maximal CMAP amplitude is critical for ensuring reproducibility and preventing overstimulation, which can cause current spread or fatigue artifacts [5, 6].

Furthermore, defining baseline amplitude and latency values under physiological conditions enables the identification of true regenerative improvements in studies employing nerve conduits, Schwann cell transplantation, stem cell therapy, or biomaterial‐based strategies [4, 8–12]. This standardization also enhances translational comparability with clinical ENMG protocols used in humans [2–7].

Therefore, the present study is aimed at standardizing an ENMG protocol for the intact median nerve in rats by identifying the minimal supramaximal stimulation intensity capable of evoking maximal CMAP amplitude with stable latency. By defining this physiological plateau condition, the study seeks to reduce methodological heterogeneity and provide a consistent reference parameter for future experimental investigations.

## 2. Materials and Methods

### 2.1. Animals

A total of 10 adult male Wistar rats (250–300 g) were used in this study. Animals were housed under controlled environmental conditions (22^°^C ± 2^°^C; 55*%* ± 10*%* humidity) with a 12‐h light/dark cycle and free access to food and water. All experimental procedures complied with institutional guidelines for animal care and were approved by the Ethics Committee for Animal Use (Protocol DHE‐ICB003). The Wistar strain was selected for its stable electrophysiological baseline and extensive use in previous ENMG studies [5]. Sample size was defined based on previous electrophysiological standardization studies of the rat median nerve employing similar group sizes [5, 6].

### 2.2. Experimental Design

The objective of this study was to standardize an electroneuromyographic (ENMG) protocol for the intact median nerve in rats. The median nerve was chosen for its well‐defined anatomy, mixed motor and sensory fiber composition, and translational relevance to human upper‐limb function [5, 6]. Moreover, the median nerve is one of the most frequently affected peripheral nerves in humans, being commonly involved in traumatic injuries and entrapment neuropathies such as carpal tunnel syndrome [2, 7]. Establishing normative electrophysiological parameters under physiological conditions provides essential reference values for subsequent models of peripheral nerve injury and repair.

### 2.3. Anesthesia and Surgical Exposure

Animals were anesthetized with intraperitoneal ketamine (100 mg/kg) and xylazine (15 mg/kg) to ensure deep anesthesia and stable recordings. Under aseptic conditions, a small incision (~1 cm) was made in the right axillary region to expose the brachial plexus and the proximal segment of the median nerve. The nerve was carefully isolated using blunt dissection without traction, transection, or manipulation that could alter conduction properties. The surgical field was kept moist with sterile saline throughout the procedure. Body temperature was maintained at 37°C with a thermostatic heating pad.

### 2.4. Electrophysiological Setup

Recordings were performed using a PowerLab 4/35 data acquisition system (ADInstruments, Sydney, Australia) and LabChart software. Signals were amplified and digitized at a sampling rate of 20 kHz and filtered using a 10‐Hz–10‐kHz band‐pass filter, which is consistent with standard CMAP acquisition parameters and adequate to preserve waveform morphology and latency accuracy. Animals were positioned in supine posture, and electrodes were stabilized to prevent motion artifacts.

### 2.5. Stimulation Protocol

**Figure 1:**
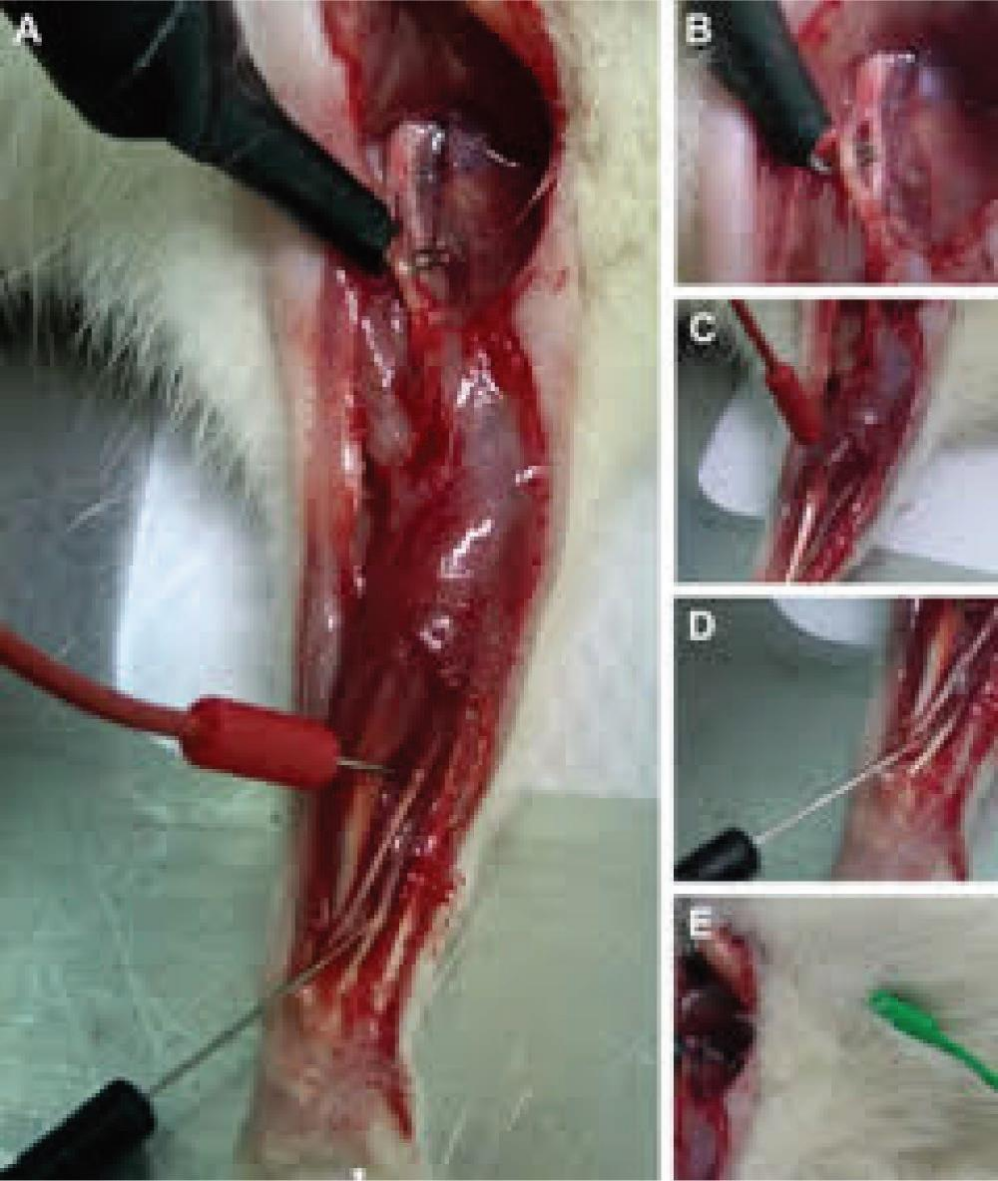
Standardized positioning of stimulation and recording electrodes for electrophysiological assessment of the rat median nerve. (A) Placement of the bipolar hook electrode and recording electrodes. (B) The bipolar hook electrode was positioned on the median nerve, with the cathode 2 mm distal to the anode. (C) The active electrode (red) was inserted into the belly of the flexor digitorum superficialis muscle. (D) The reference electrode (black) was placed over the tendon of the same muscle. (E) The ground electrode (green) was positioned subcutaneously in the right thoracic limb, near the stimulation site.

A bipolar hook electrode (ADInstruments, United States) was gently positioned underneath and in contact with the nerve at the brachial plexus level, with the cathode 2 mm distal to the anode (Figure 1B). Square‐wave pulses (0.1 ms duration, 1 Hz frequency) were applied sequentially at descending intensities of 20, 15, 10, 5, 1, 0.5, 0.25, and 0.1 V. Each stimulus was delivered three times per intensity level, and only reproducible traces with stable baselines were included for analysis. A 60‐s interval was allowed between each stimulation intensity block to minimize neuromuscular fatigue or excitability changes. The sequence was applied in descending order, beginning at 20 V, to ensure that supramaximal responses were identified before threshold‐level stimulation. All procedures were performed by the same experienced operator to reduce procedural variability.

The goal of this protocol was to identify the minimal supramaximal intensity, defined as the lowest voltage that elicited maximal CMAP amplitude without further increase at higher intensities, as previously described for peripheral nerve standardization [6].

### 2.6. Recording

Monopolar needle electrodes were used for recording CMAPs. The active electrode (red) was inserted into the belly of the flexor digitorum superficialis muscle, the primary effector innervated by the median nerve (Figure 1C). The reference electrode (black) was positioned over the tendon of the same muscle (Figure 1D). The ground electrode (green) was placed subcutaneously in the right thoracic limb near the stimulation site (Figure 1E).

This configuration minimizes crosstalk and ensures accurate muscle‐specific recordings [5]. All electrodes were disinfected between sessions and inserted at consistent anatomical landmarks under magnification.

### 2.7. Response Standardization

The standardization consisted of defining the lowest stimulation intensity capable of recruiting the largest number of muscle fibers, as evidenced by the CMAP amplitude. This ensured both the reproducibility and the sensitivity of the method while minimizing unnecessary overstimulation.

### 2.8. Parameters Analyzed

Two primary electrophysiological parameters were analyzed: (1) CMAP amplitude (mV), measured peak‐to‐peak, representing the total number of recruited motor units; and (2) latency (ms), measured from the stimulus artifact to the onset of the CMAP, reflecting conduction time along the fastest myelinated fibers.

Mean values were calculated from three consecutive traces per animal. The voltage–response relationship was plotted to determine the plateau point representing the supramaximal condition.

### 2.9. Statistical Analysis

Data were expressed as mean ± standard error of the mean (SEM). Normality was assessed using the Shapiro–Wilk test. Differences across stimulation intensities were analyzed using one‐way ANOVA or Kruskal–Wallis test when appropriate. Statistical analysis was used descriptively to support identification of the supramaximal plateau rather than to compare independent experimental groups. A *p* value < 0.05 was considered statistically significant.

## 3. Results

### 3.1. Standardization of the Electroneuromyographic Response in the Intact Median Nerve of Wistar Rats

Electroneuromyographic (ENMG) recordings were successfully obtained from all animals (*n* = 10) under stable anesthetic and temperature‐controlled conditions. All traces showed consistent waveform morphology and reproducibility across repetitions, confirming signal stability during data acquisition.

### 3.2. CMAP Amplitude

**Figure 2:**
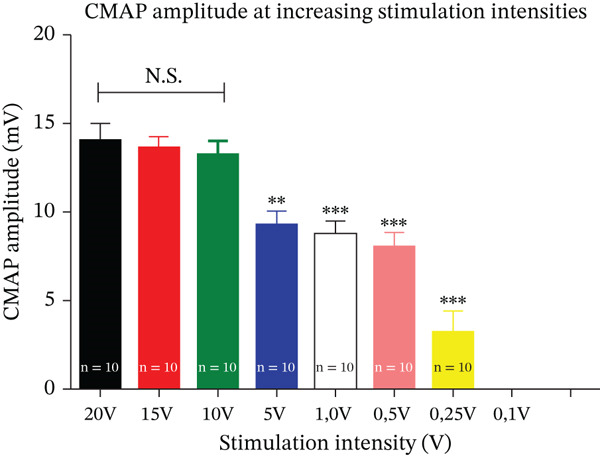
CMAP amplitude as a function of stimulation intensity. CMAP amplitude at different stimulation intensities (20, 15, 10, 5, 1, 0.5, 0.25, and 0.1 V). Data are presented as mean ± SEM.

The CMAP amplitude (Figure 2, Table 1), expressed in millivolts (mV), was analyzed across the different stimulation intensities. Stimulation between 20 and 10 V produced similar maximal responses. Stimulation intensities between 20 and 10 V produced overlapping amplitude values, characterizing a supramaximal plateau.

**Table 1:** CMAP amplitude values at different stimulation intensities. Mean CMAP amplitude (mV ± SEM) recorded at each stimulation intensity. No response was obtained at 0.1 V (NR).

Stimulation intensity (V)	Mean (mV)	Standard error of the mean	Number of animals
*20.0*	*14.05*	*0.96*	*10*
*15.0*	*13.57*	*0.69*	*10*
*10.0*	*13.24*	*0.77*	*10*
*5.0*	*9.28*	*0.76*	*10*
*1.0*	*8.70*	*0.75*	*10*
*0.5*	*8.03*	*0.80*	*10*
*0.25*	*3.23*	*1.19*	*10*
*0.1*	*—*	*—*	*10*

Amplitude decreased progressively below 10 V. At 5 and 1 V, responses were still detectable but significantly lower compared with the supramaximal range (*p* < 0.01). Minimal responses were observed at 0.5 and 0.25 V, and no muscle activity was detected at 0.1 V, confirming this value as a subthreshold stimulation.

These findings are consistent with previous ENMG standardizations in rat models, where amplitudes reached a plateau at supramaximal intensities and declined sharply below threshold [5, 6]. Based on these results, 10 V was defined as the minimal supramaximal intensity capable of eliciting maximal CMAP amplitude.

Table 1 details the mean amplitude values (± SEM) obtained from all animals at each stimulation intensity.

### 3.3. Latency

#### 3.3.1. CMAP Latency

**Figure 3:**
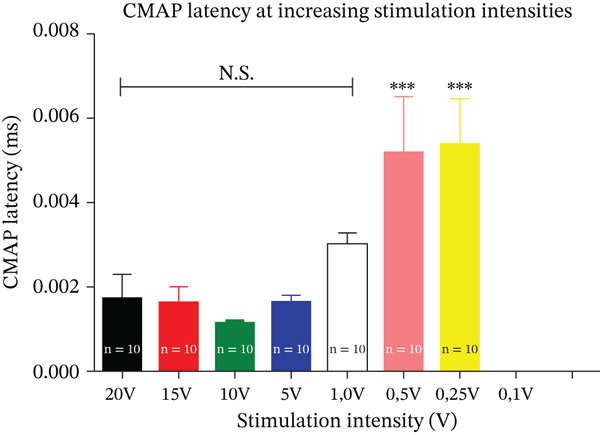
CMAP latency as a function of stimulation intensity. CMAP latency at different stimulation intensities (20, 15, 10, 5, 1, 0.5, 0.25, and 0.1 V). Data are presented as mean ± SEM.

Latency (Figure 3, Table 2), measured in milliseconds (ms), remained relatively constant between 20 and 1 V, with no significant differences across these intensities (*p* > 0.05). At 0.5 and 0.25 V, responses showed increased variability, whereas 0.1 V failed to evoke any signal.

**Table 2:** CMAP latency as a function of stimulation intensity. Mean CMAP latency (ms ± SEM) recorded at each stimulation intensity. No response was obtained at 0.1 V (NR).

Stimulation intensity (V)	Mean (ms)	Standard error of the mean	Number of animals
*20.0*	*0.00171*	*0.00059*	*10*
*15.0*	*0.00161*	*0.00039*	*10*
*10.0*	*0.00113*	*0.00014*	*10*
*5.0*	*0.00163*	*0.00017*	*10*
*1.0*	*0.00298*	*0.00029*	*10*
*0.5*	*0.00517*	*0.00132*	*10*
*0.25*	*0.00537*	*0.00109*	*10*
*0.1*	*—*	*—*	*10*

This stability of latency within the supramaximal range mirrors results reported for the rat median nerve by Casal et al. [5], where latency is preserved even as amplitude decreases at subthreshold stimulation levels.

Table 2 provides the detailed mean latency values (± SEM) obtained at each stimulation intensity.

### 3.4. Representative Traces

#### 3.4.1. Representative ENMG Traces

**Figure 4:**
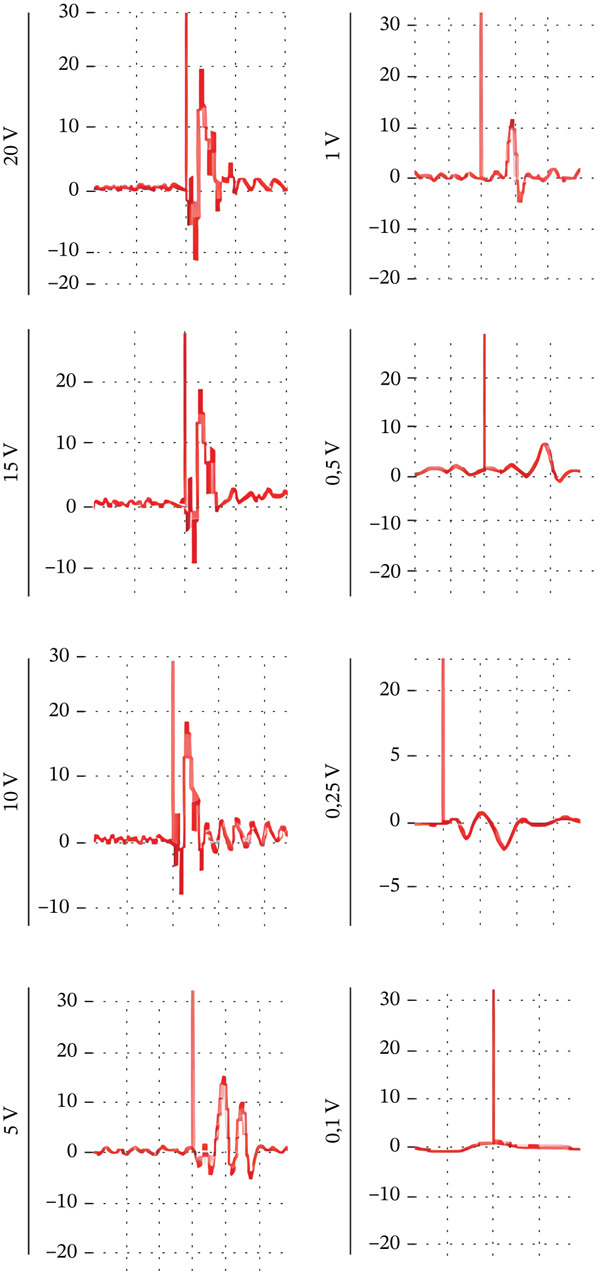
Representative ENMG recordings of the intact rat median nerve under graded stimulation. Representative ENMG traces of intact rat median nerve at 20, 15, 10, 5, 1, 0.5 V, 0.25, and 0.1 V.

Representative ENMG recordings obtained from the intact median nerve are shown in Figure 4. Distinct biphasic waveforms were observed at all supramaximal intensities, with a progressive reduction in amplitude as voltage decreased. No measurable signal was obtained at 0.1 V.

This stimulation–response relationship reproduces the typical recruitment curve pattern observed in physiological nerve conduction studies [5, 6].

## 4. Discussion

This study standardizes a supramaximal stimulation parameter (10 V) for electrophysiological assessment of the intact rat median nerve. Defining this reference intensity supports methodological consistency in experimental peripheral nerve research. [1–3].

The electrophysiological assessment of peripheral nerves is a well‐established tool for evaluating axonal and myelin function [1, 2]. Among these nerves, the median nerve is particularly suitable for experimental studies because it is anatomically accessible, exhibits a mixed composition of motor and sensory fibers, and its physiological properties are comparable across species, including rodents, primates, and humans [3–5]. Its consistent branching and identifiable muscular targets—particularly the *flexor digitorum superficialis*—enable reproducible electrode placement and response acquisition [5, 6].

The relationship between stimulus intensity and CMAP amplitude observed in this study is consistent with prior electrophysiological analyses of rat forelimb nerves, in which a plateau phase between 10 and 20 V indicated full motor unit recruitment [5–7]. At subthreshold intensities, only partial recruitment occurs, resulting in reduced amplitude and waveform variability [7, 13]. These findings confirm that the 10 V intensity effectively represents a supramaximal condition for the rat median nerve, minimizing overstimulation while ensuring complete motor activation [6, 14].

The stability of CMAP latency across supramaximal intensities also corroborates earlier studies reporting that latency remains unaffected once conduction thresholds are reached [5–7]. Since latency reflects the conduction velocity along myelinated fibers, its constancy underlines the reliability of the intact conduction pathway [1, 2]. Latency prolongation is usually seen only after demyelinating injury or chronic compression [4, 13, 14], reinforcing that the present recordings indeed reflect physiological nerve activity.

Methodological consistency in ENMG is critical for ensuring comparability across studies. Variations in electrode positioning, nerve exposure, stimulus duration, or filtering parameters can significantly alter CMAP values [5, 6, 8]. The present protocol standardizes each of these variables, allowing the generation of reproducible amplitude–intensity curves. Similar approaches have been advocated for the sciatic nerve [6] and the median nerve [5], both emphasizing that fixed electrode geometry and stimulus calibration are essential for reducing intralaboratory and interlaboratory variability.

Importantly, the results presented here establish physiological reference values for amplitude (~13–14 mV at 10–20 V) and latency (~1.1–1.7 ms), which align with previously reported parameters in uninjured rat median nerves [5, 7]. These normative data are particularly valuable for differentiating normal baseline responses from postlesional alterations in experimental repair studies using conduits [4, 8], Schwann cell transplantation [4], or stem‐cell–based constructs [9–11].

The definition of a standardized supramaximal intensity also holds translational importance. In preclinical models, supramaximal stimulation ensures that the entire nerve cross‐section is activated, reducing the risk of underestimating regeneration when assessing CMAP recovery [3, 4, 6]. In contrast, overstimulation can produce current spread to adjacent nerves or fatigue‐related variability [6, 14]. Therefore, identifying the minimal supramaximal voltage—here determined as 10 V—optimizes the balance between precision and safety, increasing reproducibility in repeated or longitudinal experiments [8, 12, 15].

Electrophysiological outcomes are also known to correlate strongly with histological and functional recovery. Casal et al. [5] demonstrated that CMAP amplitude is directly related to the number and diameter of regenerated fibers in the distal stump, whereas Archibald et al. [1, 3] and Krarup et al. [2] observed similar relationships in primate models. Moreover, Heinzel et al. [12] and Allgood et al. [15] recently expanded these findings by validating advanced ENMG configurations and digital gait analysis to evaluate functional regeneration in modern experimental setups.

Although limited to male Wistar rats, these results provide a critical first step toward a standardized electrophysiological framework applicable to both sexes and potentially to larger animal models [5, 16]. Expanding this validation to include comparative analyses of the ulnar and radial nerves, also branches of the brachial plexus, may further broaden the translational utility of this approach.

In summary, this study provides a robust methodological framework for standardized ENMG in the rat median nerve, defining 10 V as the minimal supramaximal intensity that elicits stable, reproducible CMAPs under physiological conditions. These results not only offer baseline electrophysiological parameters for intact nerves but also strengthen the foundation for translational models evaluating peripheral nerve repair, regeneration, and cortical reorganization.

Importantly, the present work is intentionally restricted to methodological standardization in intact nerves; however, defining a consistent supramaximal reference intensity has direct practical value for subsequent experimental desigsns that test regenerative or adjuvant interventions in the median nerve model. In rat median nerve repair paradigms, electrophysiological endpoints such as CMAP amplitude and latency are frequently used to quantify functional recovery after autograft and adjunct therapies, including photobiomodulation (810 nm) and other bioactive approaches, where between‐study heterogeneity in stimulation and recording settings can confound interpretation of effect sizes and comparability across laboratories [17, 18]. Similarly, translational studies in nonhuman primates have relied on combined electrophysiological and histological outcomes to support scaffold‐based strategies, reinforcing the need for harmonized acquisition parameters when bridging preclinical models and clinical neurophysiology [19]. In addition, critical appraisals of the rat median nerve injury model have emphasized that methodological inconsistencies remain a major limitation in the field and may partially explain variability in reported outcomes across repair strategies [20]. Finally, peripheral nerve injury has been associated with reorganization at central levels in primate somatosensory representations and receptive field properties, underscoring that robust and standardized peripheral electrophysiological measurements are essential for downstream studies exploring peripheral–central relationships without introducing avoidable measurement variability [16, 21]. Therefore, the standardized supramaximal stimulation parameter defined here is intended to serve as a consistent reference for future median nerve studies, improving methodological transparency and comparability across experimental contexts.

### 4.1. Limitations

This study is limited to male Wistar rats and a single stimulation site at the brachial plexus level. Additionally, quantitative intersession repeatability metrics were not assessed, as the primary aim was methodological standardization of the supramaximal intensity rather than formal reliability testing. Future studies may expand this framework by incorporating test–retest analyses and multioperator comparisons.

## Author Contributions

Tiago Bastos Taboada and Fernanda Marques Pestana contributed equally to this work.

## Funding

This study was supported by CAPES, CNPq, and FAPERJ.

## Conflicts of Interest

The authors declare no conflicts of interest.

## Data Availability

The data that support the findings of this study are available from the corresponding author upon reasonable request.
